# Multidisciplinary Exploration of Unknown Oral Lesions With Accompanying Oral Verrucas of the Tongue: A Case Report

**DOI:** 10.7759/cureus.54898

**Published:** 2024-02-25

**Authors:** Nafiz Khan, Navjot Dhoat, Ali Z Ansari, Joshua J Gallagher, Srihita Patibandla, Kurt Bruckmeier

**Affiliations:** 1 Internal Medicine, Merit Health Wesley, Hattiesburg, USA; 2 Internal Medicine, William Carey University College of Osteopathic Medicine, Hattiesburg, USA

**Keywords:** fever of unknown origin, thrombocytopenia, anemia of chronic diseases, oral infection, fever management, oral thrush, oral mucosal lesions, oral bleeding

## Abstract

A 64-year-old African American male with a history of hypertension and type II diabetes mellitus presented with unexplained upper lip lacerations after several frequent episodes of hemoptysis. Following the upper lip lacerations were several weeks of intermittent unknown episodic fevers. The patient, challenged by impaired mobility, exhibited an array of symptoms, including severe upper lip pain with lacerations and white patches on the tongue. Laboratory findings indicated thrombocytopenia and anemia, with positive tests for both influenza A and B. Despite completing Tamiflu, the patient experienced recurrent fevers. Imaging revealed gastrointestinal abnormalities, leading to the initiation of nystatin and a multi-antibiotic regimen without significant fever resolution. A subsequent tongue biopsy revealed verruca lesions, and acyclovir was initiated. Despite this, the patient developed lip and facial blisters. Negative results from cytomegalovirus (CMV) deoxyribonucleic acid (DNA) polymerase chain reaction (PCR) prompted a shift in focus to managing persistent fevers, ultimately controlled with naproxen but without discoverable cause. This case underscores the diagnostic challenge posed by unexplained fevers in an elderly patient with oral manifestations. The protracted course and evolving symptoms emphasize the intricacies of managing such cases, highlighting the need for continued investigation and collaboration across medical disciplines in navigating complex clinical scenarios.

## Introduction

Oral ulcerative lesions represent a multifaceted challenge in clinical practice, often demanding a comprehensive understanding of their etiology, differential diagnosis, and management. Among the myriad of oral mucosal disorders, recurrent oral ulcerations pose diagnostic challenges due to various causes. Careful consideration should be given to any concurrent systemic symptoms, including fever, arthritis, or other indicators suggestive of an underlying systemic condition [1]. The oral cavity is frequently overlooked in general practice examinations, yet enhanced awareness of typical oral lesion presentations can bolster practitioner confidence in conducting thorough oral examinations and effectively managing identified pathologies. It is crucial to prioritize focused oral examinations for patients with identifiable risk factors, including but not limited to smoking, solar radiation exposure, human papillomavirus (HPV) infection, and an immunocompromised state [2].

This report discusses the case of a 64-year-old African American male with a history of hypertension and type II diabetes mellitus, who presented with painful oral ulcerations and an intermittent fever of unknown origin (FUO), the persistence of which has prompted a detailed investigation into the underlying factors contributing to their occurrence. Accompanying these ulcerations are patches of oral verrucae. Verrucae represent non-malignant proliferations of the epidermis induced by human papillomavirus infection. These viral warts exhibit a predominantly outward growth pattern, displaying notable features such as hyperkeratosis and hypogranulosis [3]. Different variations can include verruca vulgaris. These oral lesions are almost always white, appear in clusters, and can grow to a maximal size or remain dormant until irritated [4]. Through a meticulous examination of the patient's medical history, treatment plan evaluation, and diagnostic tests, this report aims to unravel the complexities surrounding the etiology of these ulcerative lesions.

Fever, commonly recognized as a cardinal sign of inflammation, serves as a vital physiological response that often signifies an underlying pathological condition. The majority of febrile illnesses arise as a physiological response to disturbances within the body. Typically, these conditions either resolve before a definitive diagnosis or exhibit distinct characteristics that facilitate the diagnostic process. When fevers present without an initially obvious etiology, they are categorized as fevers of unknown origins (FUO) [5]. The conventional definition of classic FUO entails a recorded temperature exceeding 38.3°C (100.9°F) on multiple occasions over a period exceeding three weeks, with no identified cause despite thorough evaluation. Certain experts have suggested revising the temperature cutoff to exceed 38°C (100.4°F) [6]. As classic FUO patients often experience prolonged fever, many may undergo initial or sequential outpatient evaluations rather than immediate hospitalization.

Diagnosing FUO is challenging due to its diverse and complex etiologies, including infectious, inflammatory, and neoplastic causes. Diagnostic limitations, such as inconclusive laboratory results and overlapping etiologies, contribute to the difficulty in identifying the underlying cause. Patient factors, such as age, comorbidities, and medication history, must be considered alongside psychosocial implications to ensure a comprehensive evaluation. Collaborative efforts among healthcare providers and a patient-centered approach are essential for navigating the diagnostic challenges associated with FUO and achieving an accurate diagnosis. In this case report, we present insights into the patient's medical history, our evaluation plan including diagnostic tests that were performed, and steps we took in the management of FUO. This report aims to contribute to the current understanding of diagnosing FUO, taking into account patient factors, diagnostic limitations, and the importance of a patient-centered approach.

## Case presentation

The patient is a 64-year-old African American male under the care of a nursing facility, with impaired mobility and a long-term indwelling catheter, who presented to our hospital with complaints of coughing up blood. The patient is a poor historian but can answer questions and presents alert and oriented to person and place. The patient has a history of hypertension managed with amlodipine 10 mg daily and diabetes mellitus managed with metformin 500 mg daily. The patient is on pureed food, does not smoke, does not consume alcohol, and presents with no signs of self-injury, abuse, or neglect.

**Figure 1:**
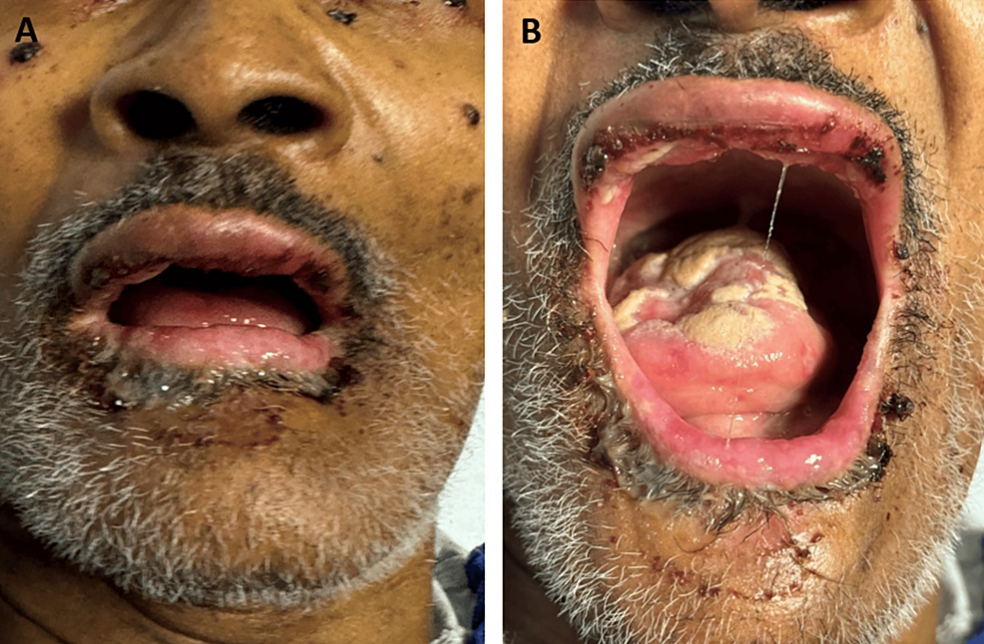
Oral lesions and lacerations noted on initial evaluation of the patient (A) Swollen upper and lower lips, and upper lip lacerations. (B) Oropharyngeal exudate with labial ulcers and verrucous lesions with plaque formation on the tongue.

On evaluation, the patient was noted to have swollen upper and lower lips, upper lip lacerations of unknown origins, and white patches on the superior aspect of the tongue (Figure 1). Neck nodes were discovered upon examination of the patient. When questioned, the patient rated his pain on the upper lip as 10/10 on the numeric pain intensity scale and described it as sharp and non-radiating. The patient was afebrile. Laboratory results indicated that his white blood cell count was 4.6 g/dL, his hemoglobin was 8.6 g/dL, and his platelet count was 42,000/μL (reference range: 164,000-446,000/μL). This was compared to the platelet count of 138,000/μL obtained at a nearby outside hospital the day prior. The patient tested positive for influenza A and B. Physical examination was otherwise unremarkable. The patient was started on Tamiflu 75 mg twice per day for five days and was admitted to the hospital.

**Figure 2:**
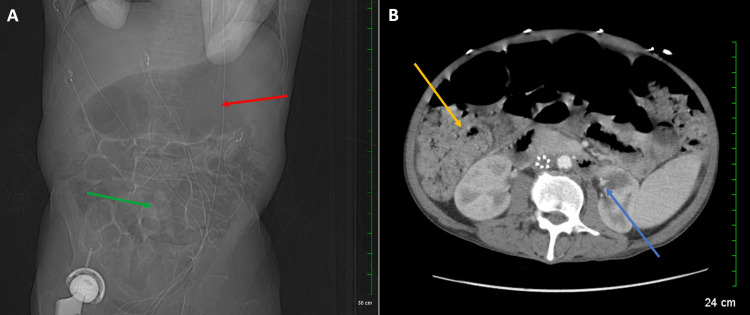
CT scan of the abdomen and pelvis with IV contrast (A) The abdomen showing an air-filled markedly distended stomach (red arrow) and a distended small bowel (green arrow). (B) The pelvis showing dilated loops of the bowel with a large amount of retained stool in the colon (yellow arrow) and a vague low-density region identified in the upper pole of the left kidney (blue arrow). CT: computed tomography, IV: intravenous

After the completion of Tamiflu, the patient presented with cyclical fever with no changes in the presentation of mouth ulcerations. His white blood count values remained unchanged from the initial, with an addition of an elevated procalcitonin level of 0.23. A computed tomography (CT) scan of the chest, abdomen, and pelvis revealed a distended small bowel, a markedly distended air-filled stomach, and an incidental vague low density in the upper pole of the left kidney (Figure 2). The patient was started on oral nystatin 400,000 units four times daily for the next 16 days. In addition to this, the patient began experiencing low blood pressure and was started on an antibiotic regime for possible sepsis from his indwelling catheter, which was last changed three weeks ago. This began with cefepime 2 g intravenous (IV) every 12 hours for three days initially. After completion and no change in presentation, the patient was given meropenem 1 g IV every eight hours. After four days of compliance and no improvement in symptoms, the meropenem was switched to amoxicillin 50 mg every eight hours. Upon having a fever spike on amoxicillin, meropenem was once again resumed for four days, and the amoxicillin was discontinued. The patient's mouth ulcerations remained unchanged, and the patient's blood counts remained the same as the initial values (Table 1).

**Table 1:** Progression of key laboratory values in accordance with diagnostic evaluation recommendations for fevers of unknown origin WBC: white blood cells, RBC: red blood cells, Hgb: hemoglobin, HCT: hematocrit, MCV: mean corpuscular volume, RDW: red cell distribution width, BUN: blood urea nitrogen

Date	11/13	11/17	11/22	Normal
WBC	5,100/mcL	7,500/mcL	5,400/mcL	4,500-11,000/mcL
RBC	3.15 × 10^6^/mcL (low)	3.20 × 10^6^/mcL (low)	2.88 × 10^6^/mcL (low)	4.35-5.65 × 10^6^/mcL
Hgb	9 g/dL (low)	9.2 g/dL (low)	8.2 g/dL (low)	13.5-17.5 g/dL
HCT	27.6% (low)	28.5% (low)	25.8% (low)	40%-54%
MCV	87.8 fL	89.2 fL	89.8 fL	80-100 fL
Platelet count	180,000/mcL	166,000/mcL	178,000/mcL	150,000-450,000/mcL
RDW	16 (high)	16.4 (high)	16.3 (high)	12-15
BUN	25 (high)	24 (high)	N/A	7-20

**Figure 3:**
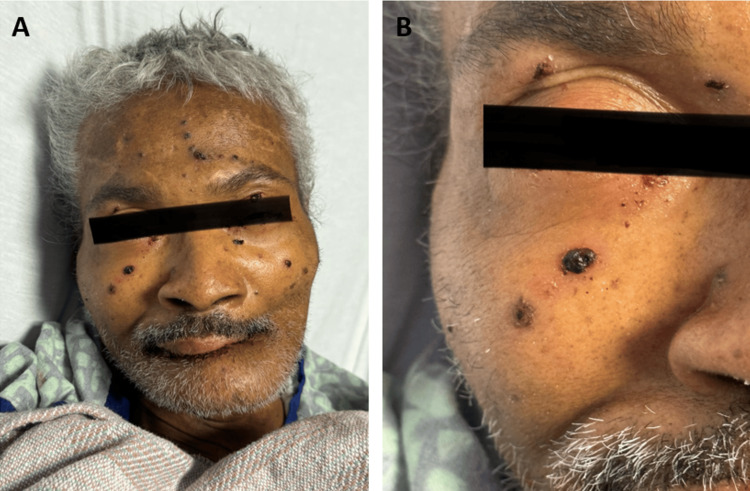
Lip and facial blisters that developed after completion of a course of meropenem (A) Scabs and ulcerated dark papules, some of which appear inflamed, scattered throughout the patient's forehead and cheeks. (B) Blisters surrounding the right eye, one of which had burst right below the patient's inner eye.

**Figure 4:**
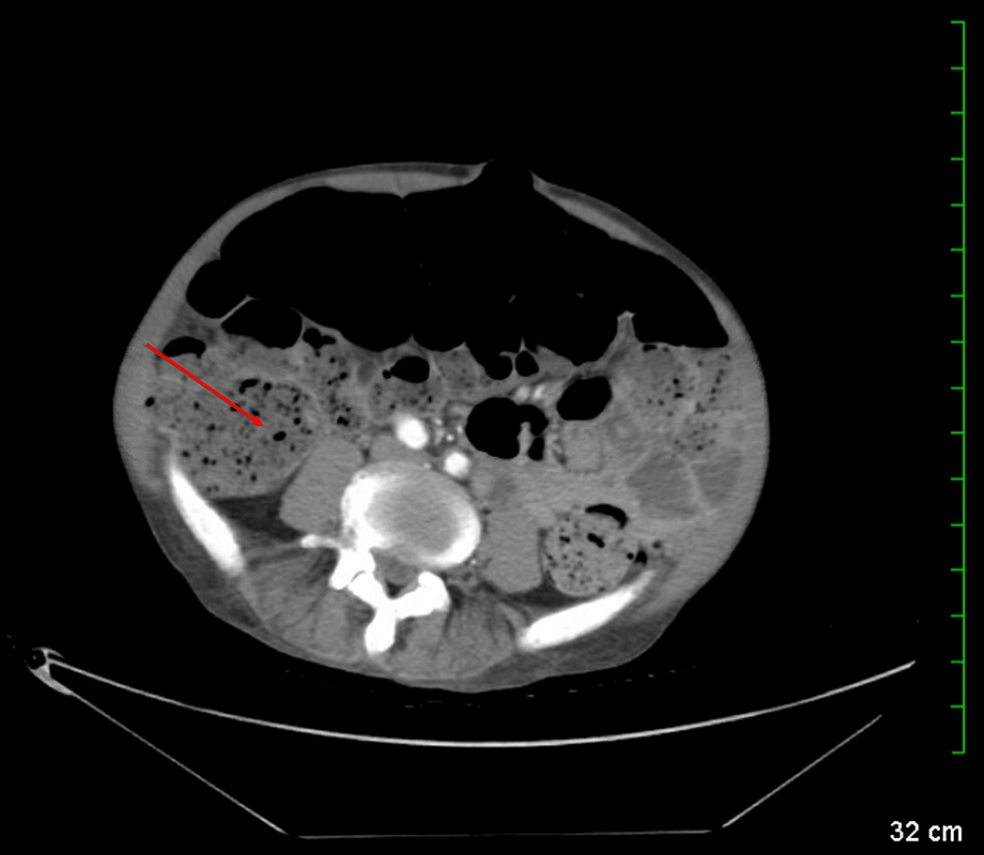
Comparative CT scan of the abdomen and pelvis with IV contrast CT demonstrating impaction with a large amount of stool within a distended colon (red arrow). CT: computed tomography, IV: intravenous

After completion of meropenem, the patient underwent a microlaryngoscopy with a tongue biopsy. Biopsy revealed traumatic verruca on the anterior and posterior tongue. Approximately, over the next three days, the patient developed lip and facial blisters that burst (Figure 3). A cytomegalovirus (CMV) deoxyribonucleic acid (DNA) polymerase chain reaction (PCR), used to check for a virally active infection, produced negative results. Despite clinical indications warranting a bone marrow biopsy for disease evaluation and monitoring, the patient adamantly refused the procedure, citing personal reasons and anxiety regarding the invasiveness of the test. The next day, the patient was started on acyclovir 400 mg IV every 12 hours and 400 mg orally every eight hours. Improvement was noted in the patient's facial lesions; however, the fevers persisted. A comparative CT scan revealed constipation (Figure 4). Infectious disease was consulted eight days later, and per their recommendation, the patient was started on naproxen, which helped resolve his cyclical fevers. A speech evaluation was performed, and no aspiration-like events were noted. The source of the patient's fever remained unknown, but he was clinically stable for discharge under hospice care as requested by the patient's power of attorney and instructions to follow up with his primary care physician.

## Discussion

The exploration of fevers of unknown origin (FUO) stands as a pivotal undertaking in clinical medicine, bearing implications for diagnosis, treatment, and public health. The complexity of these cases, exemplified by the presence of unknown facial lesions, following a persistent FUO, underscores the importance of a systematic and thorough approach.

While the infectious disease consultation suggested hepatic origins for the mouth ulcers, a definitive diagnosis has yet to be reached. The intricacy of the issue arose from the presence of a multitude of influencing factors. A differential diagnosis included infectious, immunosuppressive, or hematological etiologies. While oseltamivir did help combat the influenza virus, the patient developed cyclical fever and persistent mouth ulcerations. Further investigation through microlaryngoscopy and tongue biopsy identified verruca on the tongue yet failed to explain the entirety of the clinical presentation. Nystatin was used to treat any fungal origins, while subsequent antibiotics were used to combat various bacterial origins. Subsequent development of lip and facial blisters, despite a negative CMV DNA PCR, prompted the initiation of acyclovir. However, the patient's condition persisted, with a comparative CT revealing constipation, potentially secondary to the antibiotics. Infectious disease consultation led to the initiation of naproxen, which proved effective in controlling cyclical fevers, but no direction was given as to the origins of either condition.

A minimum of three weeks of unexplained febrile episodes is typically required to meet most proposed definitions of FUO. According to studies, up to 50% of patients with classic FUO remain without an etiologic diagnosis after extensive evaluation [7]. Patients who persistently elude diagnosis despite exhaustive evaluation exhibit a favorable prognosis, underscoring the imperative for a comprehensive and meticulous diagnostic investigation.

Regardless of the clinician's specific delineation of FUO, a systematic and methodical approach to evaluation is strongly advised. The pace of evaluation is a critical factor to consider. Patients diagnosed with classic FUO, characterized by prolonged fever, may benefit from initial or sequential outpatient assessments rather than immediate hospitalization [8]. In the case discussed, the patient's residential status at a nursing home necessitated hospitalization. Home care guidelines dictated that the patient could not return home until achieving stability and a state of being afebrile. This circumstance highlights the interplay between clinical considerations and logistical constraints in managing FUO cases, particularly for patients with specific residential requirements [9].

Fever management and source discovery involve looking into not only the patient's current presentation but also past medical history as well as social history. In the evaluation of FUO, a comprehensive diagnostic approach includes a series of blood tests such as complete blood count, complete metabolic panel, liver biochemical tests, and assessments for infectious diseases (e.g., human immunodeficiency virus (HIV) and malaria) and autoimmune conditions (e.g., antinuclear antibodies and rheumatoid factor) [10]. The inclusion of test-specific illnesses such as mononucleosis, malaria, tuberculosis (TB), or infective endocarditis is contingent on clinical suspicion. Notably, the necessity for HIV testing is determined by risk assessment. The patient's laboratory results, PCR, and toxoplasma serology did not indicate the presence of mononucleosis syndrome.

Tuberculosis is among the top three causes of FUO in the United States [11]. The tuberculin skin test (TST) and interferon-gamma release assays (IGRA) are commonly used to screen for tuberculosis (TB). However, these tests lack sensitivity and specificity, particularly in patients with any form of active TB. Although a positive result increases the likelihood of active TB, it does not confirm the diagnosis, which led to the addition of a chest X-ray during the patient's workup to rule out pulmonary TB. The patient presented negative for TB exposure.

In addition to the abovementioned workup, two sets of blood cultures, urinalysis with reflex to urine culture, and imaging via CT of the chest, abdomen, and pelvis with contrast are integral components of the investigation.

The urinary analysis of the patient indicated a significant presence of urinary bacteria (4+), a substantial number of white blood cells (too numerous to count), and moderate blood while testing negative for nitrites and bilirubin. Although urinary tract infections (UTIs) commonly manifest with symptoms such as dysuria or urinary frequency, it is noteworthy that fever may occasionally be the sole presenting sign, prompting suspicion. However, considering the patient's prior administration of cefepime, meropenem, and amoxicillin, the probability of a UTI was considered minimal [12]. Instead, we contemplated the possibility of the patient being on chronic Foley catheterization and opted against obtaining urinary cultures from the catheter source, anticipating potentially positive results attributable to colonization. Results showed no evidence of an infection localized to the pelvic region. Although malaria was initially considered in the evaluation of the patient, the absence of a travel history led us to forego further investigation in this direction [13].

The pathogenesis of multiple myeloma (MM) arises from the neoplastic proliferation of plasma cells leading to the production of a monoclonal immunoglobulin [14]. Clinical manifestations encompass bone pain with detectable lytic lesions on routine skeletal imaging, elevated total serum protein, and/or the presence of monoclonal protein in urine or serum. Additional indicators include unexplained anemia, hypercalcemia, and acute kidney failure with bland urinalysis. The definitive diagnosis is established through a bone marrow biopsy.

Multiple myeloma (MM) was indeed contemplated in our patient, prompting a thorough investigation that included a gamma gap assay and electrophoresis to discern any aberrations. Despite minor, transient elevations in ferritin, the patient's renal function exhibited overall stability throughout his hospitalization, negating the need for a definitive diagnosis.

This thorough battery of tests aimed to systematically explore potential infectious, autoimmune, and neoplastic causes of fever, providing a comprehensive overview for clinicians in the pursuit of identifying the underlying etiology of FUO. However, despite the best efforts of multidisciplinary collaboration, the etiology remains unknown. The intricacies of this case highlight the challenges in diagnosing and managing oral ulcerations in the context of a complex clinical picture, involving viral, fungal, and bacterial components. Moving forward, continued multidisciplinary collaboration and vigilant monitoring will be crucial for discovering the etiologic cause of this case and tailoring the ongoing management strategy to address the patient's unique presentation. Close follow-up and further investigations may provide additional insights into pathophysiology, ultimately guiding targeted interventions for the patient's improved well-being.

Through advancements in diagnostic methodologies, the etiology of fever often emerges within a three-week timeframe from the onset of symptoms, thereby limiting the designation of FUO to more perplexing cases. A notable enhancement to the diagnostic algorithm is the early utilization of 18F-fluorodeoxyglucose positron emission tomography/computed tomography (18F-FDG PET/CT) [15]. For patients presenting with vague symptoms and signs, utilizing 18F-FDG PET/CT as an initial diagnostic tool can effectively identify and rule out diseases, guide subsequent diagnostic steps, and prevent unnecessary invasive procedures, thus establishing its significance as a primary screening test for individuals with fever of unknown origin.

## Conclusions

Despite sequential therapeutic interventions, including antiviral medications (Tamiflu and acyclovir), oral nystatin for fungal elements, and a diverse antibiotic regimen, the persistence of oral ulcerations and the absence of marked improvement in blood counts prompted further investigations. The workup for FUO typically involves a systematic approach including history taking, physical examination, laboratory tests, malignancy screening, imaging studies, and specialized tests such as bone marrow biopsy or lumbar puncture. The identification of constipation on comparative CT raised gastroenterological considerations, highlighting the importance of exploring potential gastrointestinal contributions to the overall clinical picture. The negative CMV DNA PCR results ruled out an active viral infection, emphasizing the need for cautious interpretation of diagnostic findings. FUO may arise from a diverse array of infectious, inflammatory, neoplastic, and miscellaneous conditions, including bacterial infections such as endocarditis, autoimmune diseases such as systemic lupus erythematosus, and malignancies such as lymphomas. This case not only highlights the complexity of diagnosing oral ulcerative lesions in an elderly individual but also underscores the importance of continuous reevaluation and adaptation of therapeutic strategies. Further research into the intricate connections between oral manifestations and systemic conditions is warranted to enhance our understanding and refine clinical management approaches for similar challenging cases.
